# Observational study on the consumption of recreational drugs and alcohol by Swiss travelers

**DOI:** 10.1186/1471-2458-14-1199

**Published:** 2014-11-21

**Authors:** Céline Klunge-de Luze, Serge de Vallière, Blaise Genton, Nicolas Senn

**Affiliations:** Department of Ambulatory Care and Community Medicine, Travel clinic, University of Lausanne, Bugnon 44, 1011 Lausanne, Switzerland; Department of Medicine, Service of Infectious Diseases, University Hospital, Bugnon 44, 1011, 1011 Lausanne, Switzerland; Swiss Tropical and Public Health Institute, University of Basel, Socinstrasse 57, 4002 Basel, Switzerland

**Keywords:** Travel, Alcohol, Drugs, Recreational drugs, Risk, Behavior, Trip

## Abstract

**Background:**

Studies carried out on specific travelers’ groups such as students describe an increase in the consumption of alcohol and drugs during travel and vacation time. The present study investigates the risk behaviors (alcohol and drugs) in a general adult population in Switzerland travelling abroad who visited a travel clinic before departure.

**Methods:**

This retrospective study was conducted in a travel clinic between January 2006 and December 2008. 14,496 patients came to the clinic for a pre-travel consultation. 3,537 of them answered a questionnaire about their life habits in Switzerland and during their last trip. The only exclusion criterion was an age inferior to 18 years old.

The consumption habits of drugs and at-risk alcohol intake (8 standard drinks (SD) per week for women and 15 SD for men) was analyzed according to gender, sex, destination and profession. Predictors of adopting a risky behavior between habits in Switzerland and during their previous trip were also analyzed.

**Results:**

7% (229/3477) of participants declared having at-risk alcohol consumption in Switzerland and 14% (473/3275 [95% CI 13–16]) during their trip. 9% (332/3527) of the participants used drugs in Switzerland and 5% (178/3481) during their trip. Risk factors for at-risk alcohol consumption during a trip were: at-risk alcohol consumption in Switzerland (OR 31[95% CI 21–45]), smoking (1.7 [95% CI 1–2]), use of drugs in Switzerland (OR 2.2 [95% CI 2–3]), leisure travel (OR 1.6 [95% CI 1–2]) and managerial professions (OR 1.8 [95% CI 1–3]). Risk factors for the use of drugs during a trip were: alcohol consumption in Switzerland (OR 2.1 [95% CI 1–4]), smoking (OR 1.9 [95% CI 1–3]), and use of drugs in Switzerland (OR 29.7 [95% CI 19–45]).

**Conclusions:**

At-risk alcohol consumption and, to a lesser extent, use of drugs, affect a large number of travelers which expose them to health problems during a trip. Exploring the alcohol and drugs consumption patterns of people visiting a travel clinic should be part of the pre-travel routine consultation and would allow to identifying people who would benefit most from a specific prevention.

## Background

Swiss citizens took 10,5 million trips abroad in 2009, mostly in Europe. About 10% of the departures had an extra-European destination [1]. There are no specific recommendations or counseling during pre-travel consultations about the risks linked to the consumption of alcohol and drugs during a trip, whereas prevention against infectious diseases, road hazards or sun exposure is common [2]. Yet studies carried out on young students and travelers have demonstrated an increase in the consumption of alcohol and drugs during travel and vacation time [3–6]. Such behaviors can have significant health implications and can notably lead to road traffic accidents [7] and the appearance or aggravation of psychiatric disorders (depressive state, panic disorder, anxiety disorder, psychosis) [8]. Some behaviors can also increase the risk of arrests and detentions [9]. Moreover, a review of several studies carried out on young people under the age of twenty has shown that the consumption of alcohol was linked to an increase in unprotected casual sex and multiple or occasional sexual relationships [10, 11], which multiply the risk of contracting a sexually transmitted infection (STI).

The epidemiology and the modes of alcohol and drugs consumption are well known in Switzerland. In 2007, 15% of the population of age 15 and above abstained from drinking alcohol, 26% drank less than once a week, 44% drank every week but not every day and 14% drank on a daily basis. Women were twice more abstinent than men (21% versus 10%). The proportion of persons who drank on a daily basis increased up to the 65–74 years old group before stabilizing. The proportion of persons who had a “binge drinking” episode (more than 4 drinks per occasion for men and 3 for women) at least once a month reached its peak in the 15–24 years old group (21%) before decreasing linearly with age (2% in the group of 75 years old and above) [12]. The consumption of drugs is more frequent among young people and men, with cannabis being the most consumed drug, followed by cocaine and ecstasy [13]. According to the Swiss Health Survey of 2007, 22% of persons under 70 years old had smoked cannabis at least once in their lives, mostly among young people: 45% of men and 26% of women between 25 and 34 years old. In Switzerland, 4% of men versus 2% of women older than 15 years had used cocaine once and 2% of men versus 1% of women had tried ecstasy [14].

The information on at-risk alcohol consumption and use of drugs during travel time were derived from studies that were carried out only in specific populations or specific destinations. A recent prospective study conducted on British college students describes an increase in the number of consumers (RR=1.59) and in the frequency of alcohol consumption, as well as in the number of cannabis consumers (RR= 1.35) during the summer break [3]. Among young backpackers (18–35 years old) on a long duration trip in Australia, more than a quarter consumed alcohol and cannabis more frequently (while there was a decrease of the consumption of other drugs) [4]. In tourist resorts with significant nightlife activities, the total number of travelers who consume drugs diminishes (except for ecstasy), but those who routinely use drugs increase their consumption [5]. To our knowledge, no study has been carried out in a general population. It remains unknown to what extent such behaviors change between home and during a trip, nor which factors could predict a change of behavior. This is the aim of this study, which investigates risk behaviors (alcohol and drugs) in an adult population in Switzerland and travelling abroad who consulted a travel clinic before departure. Improving the knowledge on these specific risky behaviors may help to better target the recommendations given to travelers.

## Methods

### Population and setting

This study was conducted at the Travel Clinic of the Department of Ambulatory Care and Community Medicine of the University of Lausanne between January 2006 and December 2008. The objective was to explore through a self-administered questionnaire the life habits in Switzerland and during the last trip abroad of people visiting the Clinic. These data have been collected along a randomized clinical trial (RCT), which evaluated the impact of a brief intervention (BI) on the prevention of unprotected casual sex during travel. The method and results of this trial have been published in *BMC infectious diseases*[15]. But in brief, among all travelers filling the self-administered questionnaire, 1681 patients aged 18–44 years old planning to travel alone were randomly allocated to one of the three arms: 1) BI + provision of a box of condoms; 2) provision of a box of condoms alone and 3) usual care. The two interventions were provided by trained staff of the travel clinic. A post-travel questionnaire was also filled by these patients in order to assess the efficacy of the interventions by measuring the rate of unprotected casual sex intercourses during the trip.

Patients of the travel clinic are seen by nurses or physicians under the supervision of travel medicine specialists. A standard consultation lasts from 15 to 25 minutes. About 7,000 travelers are seen yearly at the Clinic on one or more occasions.

### Data collection

Between January 2006 and December 2008, 14,496 eligible travelers visited the Clinic for a pre-travel consultation. Among them, 3,537 participants were recruited by the administrative staff in the waiting room. The only exclusion criterion was an age inferior to 18 years old. The limited number of administrative personnel explains the low rate of participants in comparison with the total number of clients. All the participants filled in a self-administered questionnaire in the waiting room. It concerned their life habits in Switzerland and during their previous trip (last trip out of Switzerland). The questions related to demographic characteristics (gender, origin, marital status, occupation), their sexual habits (sexual orientation, spouse, occasional partner(s), protection during occasional sexual intercourse), their risk behaviors in Switzerland and abroad (consumption of alcohol, use of drugs and unprotected casual sex with an occasional partner), and the purpose of their last trip (vacation-leisure, business trip).

### Study design and procedure

This is an observational study using retrospective data, whose goal is to describe the risk behaviors of adults in Switzerland and during their last trip abroad: at-risk alcohol consumption and use of drugs. At-risk alcohol consumption has been defined as being superior or equivalent to 8 standard drinks (SD) per week for women and 15 SD for men. This conservative definition is broadly used not only in Switzerland [16] but also internationally. It is indeed recommended by the *National Institute on Alcohol Abuse and Alcoholism* in the United States and taken as reference in many scientific publications [17]. These low thresholds were also used because we were mostly interested in investigating changes of habits between home and travel rather exploring absolute consumption. Therefore lower thresholds were more likely to capture changes of behaviors. With regard to drugs, we considered that any consumption was at-risk, regardless of the substance or the quantity (cannabis, ecstasy, cocaine, LSD, NMDA, heroin, speed, hallucinogenic mushrooms).

The risk behaviors were analyzed according to the following variables: 1) age category (<25 years old, 25–35 years old, 36–45 years old and >45 years old); 2) gender; 3) occupation, divided in 5 groups depending on the socioeconomic status (A: academic and intellectual professions, B: managers and universities of applied sciences (HES), C: Federal Certificate of Proficiency (CFC) and employees, D: retired and unemployed persons and E: students) in accordance with the *International Standard Classification of Occupations* from the International Labor Organization *(ISCO-88)*; 4) countries of destination classified by regions of the world according to the United Nations (Africa [AF], America [AM], Asia [AS] and Europe [EU]; Oceania [OC] and multiple destinations [MD] were not taken into account given the low number of travelers) [18, 19] and 5) type of travel (vacation-leisure [visiting family or friends] vs. business trip). The predictive factors of a change of behavior were also investigated (new risk behavior during the trip in comparison with behavior at home).

The study was approved by the ethics committee of the University of Lausanne in September 2005 and registered (ClinicalTrials.gov: NCT01056536).

### Data analysis

The data were entered in Access software (Microsoft Office 2003) and the statistical analyses were performed on Epi Info 3.5.3. and STATA 12.0. A chi^2^ test was used to investigate the difference observed between two proportions (p < 0.05). A bivariate analysis was performed to calculate odds ratios (OR) and to identify the predictive factors of risk behavior during a trip and of a change of behavior during a trip with a confidence interval of 95%. The predictive factors with an OR where the confidence interval did not include 1 in the bivariate analysis were taken into account and entered into an analysis for the purpose of building a multivariate logistic regression model in order to calculate the OR adjusted to the predictive factors of the cohort.

## Results

### Description of the cohort

**Figure 1:**
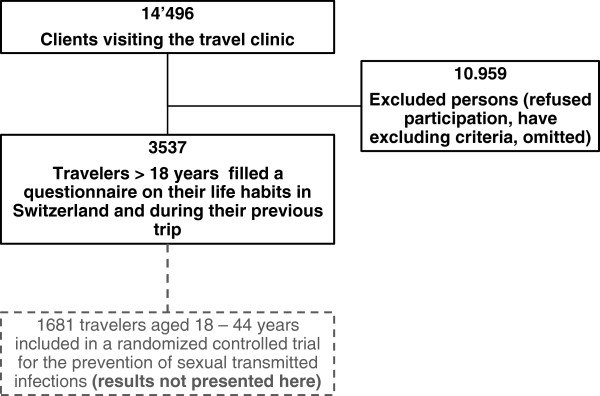
**Study flow-chart.**

Among the 14,496 eligible travelers who visited the travel clinic during the study, 3,537 filled in the first questionnaire. 1,681 persons took part in the randomized study on the BI and completed the second questionnaire when they came back from their trip. Figure 1 displays the study flowchart.

Table 1 summarizes the general characteristics of all the participants among whom 72 % were Swiss, 17% came from Europe (outside of Switzerland), 4% from Africa, 3% from America and 1% from Asia.

**Table 1:** **General characteristics of the participants**

Characteristics		Percent (%)	95% IC
N	3537		
Female	1771	50	48-52
Mean age (years)	34		
Nationality Swiss	2557	72	71-74
**Destination previous trip**			
Africa	558	16	15-17
America	649	18	17-20
Asia	582	16	15-18
Europe	1524	43	41-45
Oceania	64	2	1-2
Multiple destinations	27	1	1
Others	133	4	3-4
**Main reason for trip**			
Leisure	3056	86	85-88
Professional	393	11	10-12
Unknown	88	3	2-3
**Professional categories**			
A (academics and intellectuals)	730	21	20-23
B (managers)	1018	30	28-31
C (employees)	858	25	24-26
D (retired and unemployed)	147	4	4-5
E (students)	686	20	19-21

### Prevalence rates of risk behaviors in Switzerland vs. during the previous trip

**Figure 2:**
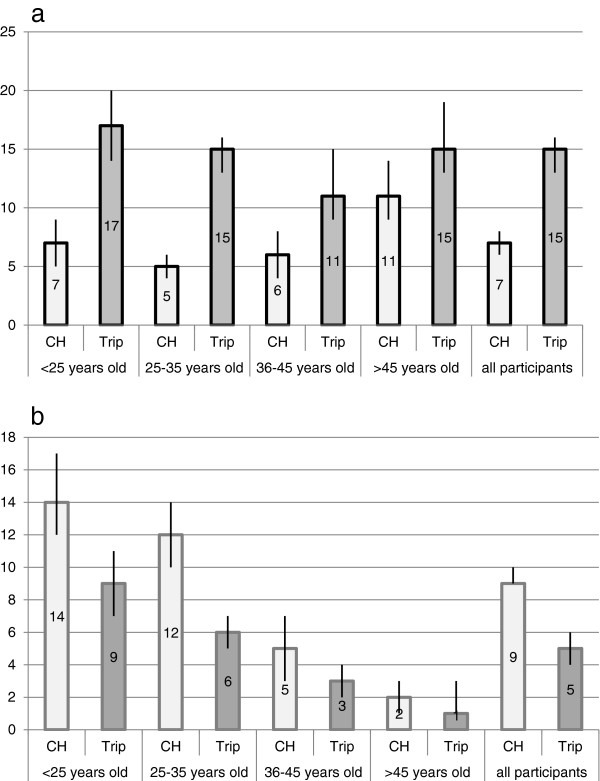
**At-risk consumption of alcohol (a) and use of drugs (b) in Switzerland and during their last trip by group of age (prevalence in percent).**

In Switzerland, 56% (1980/3529 [95% IC 55–58] of the participants drank alcohol, their average consumption being 6.1 SD per week. During the trip, 67% (2327/3481 [95% IC 65–68]) of the participants drank alcohol and their average consumption was 8.1 SD per week (p < 0.01). At-risk alcohol consumption in Switzerland was reported by 7% (229/3477 [95% IC 6–8]) of the participants. During the trip, 14% (473/3275 [95% IC 13–16]) of the participants had at-risk consumption (p < 0.01).Nine percent (332/3527 [95% IC 9–10]) of the participants used drugs in Switzerland and 5% (178/3481 [95%CI 4–6]) while travelling. Cannabis was the most consumed drug in both cases: 86% (285/332) in Switzerland and 87% (154/178) during the trip. The other drugs consumed in Switzerland were: cocaine (N = 23), ecstasy (N = 18), LSD (N = 7), MDMA (N = 5), heroin (N = 2), speed (N = 2) and hallucinogenic mushrooms (N = 1). Figure 2 displays the prevalence rates in Switzerland and abroad of at-risk alcohol consumption (2a) and drugs use (2b) stratified by age categories.

### Risk behaviors according to gender in Switzerland and during a trip

**Figure 3:**
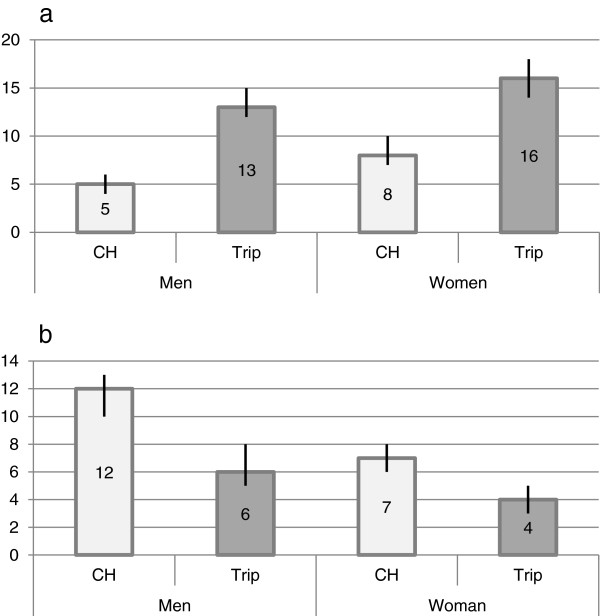
**At-risk alcohol consumption (a) and drug use (b) by gender (prevalence in percent).**

In Switzerland, 49% (864/1770 [95% CI 47–51]) of women drank alcohol and their average consumption corresponded to 4.9 SD per week. As far as men were concerned, 64% (1111/1749 [95% CI 61–66]) of them drank alcohol with an average consumption of 7.1 SD per week. In Switzerland, 17% (144/840 [95% CI 15–20]) of women who drank had at-risk consumption compared to 8% of men who drank (85/1084 [95% CI 6–10]). During a trip, 61% (1058/1744 [95% CI 58–63]) of women drank alcohol and their average consumption corresponded to 6.4 SD per week, whereas 73% (1263/1727 [95% CI 71–75]) of men drank alcohol with an average consumption of 9.6 SD per week. Figure 3 shows the prevalence rates of at-risk alcohol consumption (3a) and drug use (3b) stratified by gender.

### Risk behaviors according to destination and purpose of trip

Nineteen percents (268/1436 [95% CI 17–21]) of the participants had at-risk alcohol consumption during a trip in Europe, 13% (77/604 [95% CI 10–16]) in America, 10% (53/518 [95% CI 8–13]) in Africa and 11% (61/554 [95% CI 9–14]) in Asia. Six percents (83/1522 [95% CI 4–7]) of the participants consumed drugs in Europe, 6% (39/647 [95% CI: 4–8]) in America, 5% (25/558 [95% CI: 3–7]) in Africa and 3% (18/579 [95% CI: 2–5]) in Asia.

During a vacation-leisure trip (VL), 15% (428/2865 [95% CI 14–16]) of the participants had at-risk alcohol consumption and 11% (41/371 [95% CI 8–15]) during a business trip (BT).

During a VL, 5% (162/3046 [95% CI 5–6]) of the participants used drugs and 4% (15/393 [95% CI 2–6]) during a VP.

### Predictors of risk behaviors during a trip

Table 2 presents odds ratios (OR) of potential risk factors for travelers who engaged in a risk behavior (at risk alcohol consumption or drugs use) during their last trip (N = 3537) in bivariate analysis.

**Table 2:** **Predictors of risk behavior during a trip**

	Drug consumption			At-risk alcohol consumption		
Characteristics	N	Drug	OR (95% CI)	N	Alcohol	OR (95% CI)
Women	1747	67 (4%)	0.6 (0.4-0.8)	1664	264 (16%)	1.3 (1-1.5)
<36 years old	2235	153 (7%)	3.9 (2.5-6.3)	2105	317 (15%)	1.1 (1-1.4)
Smoker	975	123 (13%)	6.4 (4.6-8.9)	908	216 (24%)	2.6 (2.1-3.1)
Drug use in Switzerland	322	133 (41%)	50 (34.3-72)	297	94 (32%)	3.2 (2.4-4.1)
At-risk alcohol consumption in Switzerland	227	30 (13%)	3.2 (2-4.9)	216	169 (78%)	33 (23.3-46.5)
Without stable partner	1051	72 (7%)	1.6 (1.2-2.2)	988	166 (17%)	1.3 (1.1-1.6)
**Type of travel**						
Business trip	393	15 (4%)	0.7 (0.4-1.2)	371	41 (11%)	0.7 (0.5-1)
Vacation-leisure	3046	162 (5%)	1.4 (0.8-2.4)	2866	429 (15%)	1.4 (1-2)
**Professional category**						
A (academics and intellectuals)	N/A	N/A	Ref.	N/A	N/A	Ref.
B (managers and HES)	1010	46 (5%)	1.5 (0.9-2.4)	955	163 (17%)	1.6 (1.2-2.2)
C (employees and CFC)	836	48 (6%)	1.9 (1.1-3.1)	780	105 (13%)	1.2 (0.9-1.7)
D (retired and unemployed)	143	3 (2%)	0.7 (0.2-2.2)	130	19 (15%)	1.4 (0.8-2.3)
E (students)	672	54 (8%)	2.7 (1.6-4.4)	634	94 (15%)	1.4 (1-1.9)

In multivariate analysis, the following predictors were associated with a risk behavior during a trip. With regard to at-risk alcohol consumption during a trip: at-risk alcohol consumption in Switzerland (OR 30,8 [95% CI 21–45]), smoking (OR 1.7 [95% CI 1–2]), use of drugs in Switzerland (OR 2.2 [95% CI 2–3]), leisure travel (OR 1.6 [95% CI 1–2]) and professional category of managers (OR 1.8 [95% CI 1–3]). With regard to use of drugs during the trip: consumption of alcohol in Switzerland (OR 2.1 [95% CI 1–4]), smoking (OR 1.9 [95% CI 1–3]) and use of drugs in Switzerland (OR 29.7 [95% CI 19–45]).

### Predictors of a change of behavior (switching to a risk behavior during a trip)

The total number of people who engaged in a new risk behavior during their vacation corresponds to 298/473 (63%) with regard to alcohol and to 44/178 (25%) with regard to drugs. Table 3 presents the predictors of a change of behavior during a trip.

**Table 3:** **Predictors of a change of behavior during a trip**

	Drug consumption			At-risk alcohol consumption		
Characteristics	N	Drug	OR (95% CI)	N	Alcohol	OR (95% CI)
Women	1752	21 (1%)	0.9 (0.5-1.7)	1669	155 (9%)	1.1 (0.8-1.3)
<36 years old	2242	39 (2%)	4 (1.6-10.3)	2113	219 (10%)	1.6 (1.2-2.1)
Smoker	984	19 (2%)	2 (1.1-3.6)	917	124 (14%)	2 (1.6-2.5)
Drug use in Switzerland	N/A	N/A	N/A	300	59 (20%)	2.8 (2.1-3.9)
At-risk alcohol consumption in Switzerland	229	8 (4%)	3.2 (1.5-6.9)	N/A	N/A	N/A
Without partner	2428	25 (1%)	0.6 (0.3-1)	2287	198 (9%)	0.8 (0.7-1.1)
**Type of travel**						
Business trip	N/A	Ref.	N/A	N/A	Ref.	N/A
Vacation-leisure	3048	43 (1%)	5.6 (0.8-40.8)	2873	274 (10%)	1.8 (1.1-2.8)
**Professional catagory**						
A (academics and intellectuals)	N/A	Ref.	N/A	N/A	Ref.	N/A
B (managers and HES)	1012	10 (1%)	1 (0.4-2.7)	958	119 (12%)	2.2 (1.5-3.2)
C (employees and CFC)	841	7 (1%)	0.9 (0.3-2.5)	784	58 (7%)	1.2 (0.8-1.9)
D (retired and unemployed)	143	1 (1%)	0.7 (0.1-6)	130	8 (6%)	1 (0.5-2.2)
E (Students)	672	18 (3%)	2.8 (1.2-6.8)	637	66 (10%)	1.8 (1.2-2.7)

The adjusted OR for predictors for a change of behavior during a trip, with regard to at-risk alcohol consumption, were: smoking (OR 1.5 [95% CI 1–2]), use of drugs in Switzerland (OR 2.2 [95% CI 2–3]), leisure travel (OR 1.7 [95% CI 1–3]) and the professional category of managers (OR 2 [95% CI 1–3]); with regard to drugs: consumption of alcohol in Switzerland (OR 3.4 [95% CI 2–8]).

## Discussion

This study explores the consumption of alcohol and drugs of clients of a travel clinic during their trip abroad in comparison to their habits in Switzerland. It shows that at-risk alcohol consumption and use of drugs was a problem that travelers often encounter. Overall at-risk alcohol consumption increased during a trip (from 7% to 14%), while the use of drugs appears to decrease during a trip (from 9% to 5%).

The increase of at-risk alcohol consumption affected all of the participants. It was however stronger among young people under 35 years old (from 5% to 15%), while older people had a higher at-risk alcohol consumption at home (11%), which increased less sharply during a trip (15%). This may be explained by different habits of consumption: binge drinking is more frequent among young people, while a chronic excessive consumption mostly affects people above 55 years old [12]. Moreover, there were more women than men who had at-risk alcohol consumption at home (8% versus 5%) and during a trip (16% versus 13%), unlike the report of the Swiss Health Survey of 2007, which showed that punctual inebriations (more than 5 SD/occasion) and chronic excessive consumption (more than 4 SD/day) were more prevalent among men. The definitions of at-risk alcohol consumption [16] used in our study establish different thresholds for men and women, which partly explains these results (the absolute average consumption being lower for women: 4.9 SD/week versus 7.1 SD/week for men in Switzerland). The prevalence of at-risk alcohol consumption was significantly stronger in the case of European destinations, which is most likely linked to local habits: the consumption of alcohol in Europe is also higher in comparison with other regions of the world, according to the last *Global status report on alcohol and health* published by the World Health Organization (WHO) [20]. In addition, it may be that it is more difficult to get alcohol outside of Europe, in particular in predominantly Muslim countries.

The consumption of drugs decreased significantly during a trip, dropping from 9% to 5% of all the participants, and only 1% had a new consumption of drugs while travelling. This decline applied to all of the participants regardless of their age or gender. This may be explained by the fact that despite the worldwide production and consumption of cannabis, [21] many countries have introduced very restrictive regulation which has reduced its availability. The decrease of drugs consumption during a trip is in contradiction with the results of previous studies. It may be explained by the fact that this study pertains to a general population of travelers who are more concerned about their health since they consult a Travel Clinic before a trip and, consequently, might reduce their consumption of drugs during a trip. Moreover, persons who travel to Central Europe and Western Europe have a higher consumption of drugs than the average, which may explain the decrease of drugs consumption during a trip to other regions of the world. There is most likely a problem of accessibility in certain countries (travelers are less familiar with the market than in their own country). Furthermore, fear of foreign laws, which may be severe with regard to drugs, is likely to be an impediment to consumption [22].

Pre-travel consultation is the ideal moment to address the aspects of travel-related hazards and the consumption of alcohol and drugs should be part of it, given the prevalence of these risky behaviors. It is however often difficult to address all aspects of travel prevention, as the major part of the consultation is often dedicated to infectious diseases (malaria, diarrhea, etc.). In this context, it is useful to know the predictors of a risk behavior in terms of alcohol and drugs use in order to better target the prevention messages to travelers who need them.

In our study, we observed that people who had an excessive consumption of alcohol or who used drugs in Switzerland will continue this behavior during their trip. We also observed that smoking, travelling for leisure and the professional category of managers were also significant risk factors. Moreover, the persons who engaged in a new risk behavior during a trip by increasing their consumption of alcohol or by using drugs for the first time will put themselves in particular danger. In our study, smoking, use of drugs, travelling for leisure and being in a managerial position are predictors of a change of behavior with regard to the consumption of alcohol. Being aware of these factors can be useful in routine practice and simply asking the travelers about their consumption of alcohol and drugs would allow for the identification of those who would benefit most from receiving specific preventive advice. These aspects of prevention are an integral part of primary care consultations, but are rarely addressed during pre-travel counseling. In this respect, it is interesting to notice that the last American recommendations in travel medicine only mention alcohol as a cause of road traffic accidents [2]. Finally, we also noticed that, despite widespread misperceptions, young age and being a student are not linked to a risk behavior with regard to the consumption of alcohol during a trip.

Among the limitations of this study, first it is likely the participants are concerned about their health since they consulted a Travel Clinic and therefore represent a lower-risk profile than the general population. Second, the measure of the consumption of alcohol and drugs was performed retrospectively (after the last trip), a recall bias might therefore underestimate the true prevalence rates. However, we estimated prospectively, along the randomized controlled trial on the prevention of STI, [15] the prevalence rates of drugs and at-risk alcohol consumption in a subgroup of travelers (Klunge *et al.* unpublished data) who filled a post-travel questionnaire and found out the following prevalence rates: 18% for excessive consumption of alcohol (versus 14% during the last trip) and 7% for drugs (versus 5%). These numbers, while slightly higher in comparison with those obtained retrospectively in this study (which was expected due to recall bias) confirm the importance of the risks taken during a trip by travelers. One of the strengths of this study is the significant size of the sample, which allows a sufficient power to analyze the predictors of a change of behavior. Another factor that might have modified the true prevalence rates is the fact that it was a self-administered questionnaire. The collected data relied therefore entirely on the willingness to answer of the travelers.

Another limitation is that the questionnaire did not record binge drinking behavior. It would have been indeed interesting to have this additional information as it is now a well recognized public health problem affecting different groups of populations. One reason explaining this missing information is that when the questionnaire was created in 2005, binge drinking was less of a recognized problem than now. However, with the low thresholds used in this study (8 SD for women and 15SD for men) it is likely that we have caught most problematic alcohol consumers.

Finally, the data were collected six years ago, raising the question of knowing if it is timely enough to provide accurate conclusions. We believe however, that it is unlikely that drugs and alcohol consumption’s habits have sufficiently changed over these six years to significantly affect the results.

## Conclusions

At-risk alcohol consumption and, to a lesser extent, use of drugs, affect a large number of travelers, regardless of gender or age. Investigating the habits of alcohol and drug consumption of travelers should be an integral part of the pre-travel routine consultation and would allow for the identification of the people who would benefit most from a specific prevention. However, it remains to be determined which interventions are best suited to prevent such risk-taking during a trip. More generally, prevention messages regarding the risks associated with excessive consumption of alcohol and use of drugs during a trip should be more widely disseminated in travel clinics.

## References

[1] Stalder S, (OFS) Ofdls (2010). Travels of the resident population in 2009 [French]. Actualités OFS.

[2] Schwartz BS, LaRocque RC, Ryan ET (2012). Travel Medicine, In the clinic. Annals of Internal Medicine.

[3] Vivancos R, Abubakar I, Hunter PR (2010). Foreign travel associated with increased sexual risk-taking, alcohol and drug use among UK university students: a cohort study. Int J STD AIDS.

[4] Bellis MA, Hughes KE, Dillon P, Copeland J, Gates P (2007). Effects of backpacking holidays in Australia on alcohol, tobacco and drug use of UK residents. BMC Public Health.

[5] Bellis MA, Hale G, Bennett A, Chaudry M, Kilfoyle M (2000). Ibiza uncovered: changes in substance use and sexual behaviour amongst young people visiting an international night-life resort. Int J Drug Policy.

[6] Bloor M, Thomas M, Hood K, Abeni D, Goujon C, Hausser D, Hubert M, Kleiber D, Nieto JA (1998). Differences in sexual risk behaviour between young men and women travelling abroad from the UK. Lancet.

[7] Risquez A, Marrero A, Naranjo N, Palacios Y (2010). Diseases and injuries associated with travel among students, employees and teachers of the central University of Venezuela during the national summer vacations. Travel Med Infect Dis.

[8] Beny A, Paz A, Potasman I (2001). Psychiatric problems in returning travelers: features and associations. J Travel Med.

[9] MacPherson DWGB, Sandhu J (2007). Arrest and detention in international travellers. Travel Med Infect Dis.

[10] Cooper M (2002). Alcohol use and risky sexual behavior among college students and youth: evaluating the evidence. J Stud Alcohol Suppl.

[11] Bellis MA, Hughes K, Calafat A, Juan M, Ramon A, Rodriguez JA, Mendes F, Schnitzer S, Phillips-Howard P (2008). Sexual uses of alcohol and drugs and the associated health risks: a cross sectional study of young people in nine European cities. BMC Public Health.

[12] Delgrande Jordan M, Notari L (2011). Alcohol consumption in Switzerland: an analysis of data from the Swiss Health Survey 2007 [French].

[13] EMCDDA (2011). State of the drugs problem in Europe, 2011 Annual Report.

[14] **Swiss Health Survey** http://www.addictionsuisse.ch/infos-und-fakten/

[15] Senn N, De Vallière S, Berdoz D, Genton B (2011). Motivational brief intervention for the prevention of sexually transmitted infections in travelers: a randomized controlled trial. BMC Infectiuos Dis.

[16] Daeppen J (2003). Vade mecum of alcology.

[17] **Drinking Levels Defined** http://www.niaaa.nih.gov/alcohol-health/overview-alcohol-consumption/moderate-binge-drinking

[18] Deglon-Fischer A, Barth J, Ausfeld-Hafter B (2009). Complementary and alternative medicine in primary care in Switzerland. Forsch Komplementmed.

[19] **Standard Country or Area Codes for Statistical Use** http://unstats.un.org/unsd/methods/m49/m49.htm

[20] WHO (2011). Global status report on alcohol and health.

[21] United Nations oadac (2010). World Report on Drugs [French].

[22] Federal Department of Foreign Affairshttp://www.eda.admin.ch

[CR23] The pre-publication history for this paper can be accessed here: http://www.biomedcentral.com/1471-2458/14/1199/prepub

